# Keeping it cool

**DOI:** 10.7554/eLife.86885

**Published:** 2023-03-23

**Authors:** Vy Nguyen, Iain Searle

**Affiliations:** 1 Department of Molecular and Biomedical Sciences, School of Biological Sciences, The University of Adelaide Adelaide Australia

**Keywords:** flowering, vernalization, COOLAIR, CBF, FLC, *A. thaliana*

## Abstract

A well-established model for how plants start the process of flowering in periods of cold weather may need revisiting.

**Related research article** Jeon M, Jeong G, Yang Y, Luo X, Jeong D, Kyung J, Hyun Y, He Y, Lee I. 2023. Vernalization-triggered expression of the antisense transcript COOLAIR is mediated by CBF genes. *eLife* **12**:e84594. doi: 10.7554/eLife.84594.

The timing of flowering is one of the most important transitions during the life of a plant. Synchronising the flowering period with warmer temperatures enhances the reproductive success of a plant (and its impact on pollinators). To do so, plants must be able to sense cold temperatures, distinguish between short- and long-term periods of cold, and remember when these changes take place. This requires a complex interplay between internal regulators and environmental cues (Simpson and Dean, 2002; Wellmer and Riechmann, 2010).

In some plants, such as the model plant *Arabidopsis thaliana*, prolonged periods of cold lasting several weeks are required to initiate flowering via a process known as vernalisation. During this time, a gene called *Flowering Locus C* (*FLC*), which acts as a brake to flowering, gets switched off by epigenetic processes that stably hold the gene in the off position long after the vernalisation period (Michaels and Amasino, 1999; Sheldon et al., 1999; Michaels and Amasino, 2000; Whittaker and Dean, 2017).

Several decades of intensive research using a wide range of techniques has led to a well-established model for how the flowering block imposed by *FLC* is regulated. A long, non-coding RNA, called *COOLAIR*, is thought to play an important role in this model. Since *COOLAIR* is an antisense RNA with a complementary sequence of nucleotides to *FLC*, it is assumed to be an ideal candidate for suppressing the expression of this gene. Now, in eLife, Ilha Lee and colleagues – including Myeongjune Jeon and Goowon Jeong as joint first authors – report new results that challenge this theory (Jeon et al., 2023).

To better understand the role of *COOLAIR* in vernalisation, the researchers – who are based at the Seoul National University, the Chinese Academy of Sciences, and the Peking University Institute of Advanced Agricultural Sciences – did a combination of molecular, genetic and physiology experiments before, during and after vernalisation in *A. thaliana* plants and cells to explore the role of *COOLAIR* and some DNA transcription factors, known as CBFs, in vernalisation.

In a search for factors involved in the early stages of the vernalisation process, Jeon et al. focussed on the cold signal transduction that regulates the transcription of *COOLAIR*. The results revealed that the CBFs are required for *COOLAIR* transcription (Figure 1). CBF proteins have been known to play a central role for plants to increase their tolerance to freezing but they had not been linked to the vernalisation response before. Moreover, the CBF-binding DNA sequence in the *COOLAIR* promoter (a critical sequence for transcription) is conserved amongst different plant species, suggesting the CBF-*COOLAIR* regulator mechanism evolved some significant time ago.

**Figure 1.**
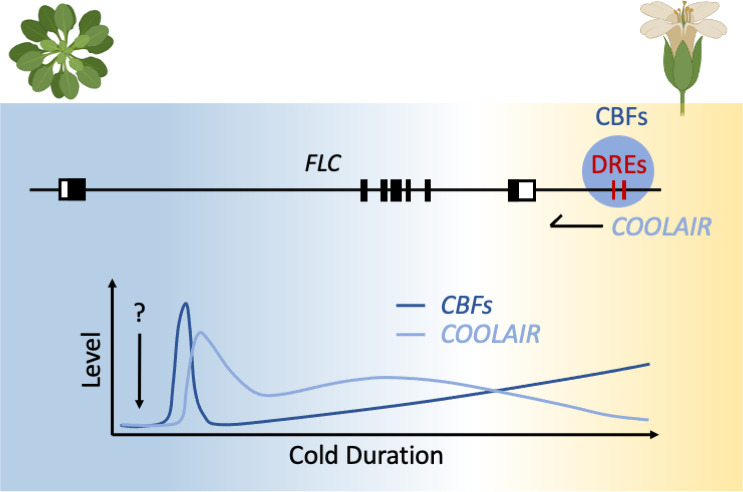
Vernalisation in *Arabidopsis thaliana*. Some plants need a prolonged period of cold lasting several weeks to permit flowering in a process known as vernalisation. In *A. thaliana*, a gene called *FLC* stops plants from flowering during winter. During this time, FLC is highly expressed (black bars), but this activity is reduced after vernalisation. Jeong et al. show that early in the vernalisation process, CBF proteins (dark blue) bind to conserved DNA sequences (DREs) at the end of *FLC* to transcribe a long non-coding RNA, called *COOLAIR* (light blue). During vernalisation, the amount of CBFs increases, while *COOLAIR* levels decrease. The upstream regulator of CBF transcription remains unknown (shown as question mark).

In genetically modified plants that only had inactive forms of the genes coding for CBFs, cold-induced expression of *COOLAIR* was severely impaired. In modified plants with overactive CBF-coding genes, *COOLAIR* levels were high even when temperatures were warm. Unexpectedly, Jeon et al. found that these genetically modified plants were still able to go through vernalisation, even though they were unable to produce *COOLAIR* during the cold period. In addition, well described epigenetic histone modifications responsible for switching off the *FLC* gene remained unchanged before and after vernalisation in the genetically modified plants. This suggest that neither *COOLAIR*, nor the CBF transcription factors that transcribe *COOLAIR*, are required to induce vernalisation.

Jeon et al. used an elegant combination of genetic and molecular experiments to paint a compelling picture that suggests that the previously well-established vernalisation model requires updating. This is also in accordance with recent research, suggesting a similar theory (Helliwell et al., 2011; Luo et al., 2019). There are only a few genes where an in-depth analysis of how they are controlled has improved our overall knowledge of gene regulation. These include the *trp* operon genes in *Escherichia coli*, which regulate synthesis of the essential amino acid tryptophan and the *beta-globin* locus in mammals, famous for their involvement in the transport of oxygen (Yanofsky, 1981; Myers et al., 1986). It is clear the *FLC* locus also deserves a spot on this list.
